# Progress of the Medical Sciences

**Published:** 1891-09

**Authors:** 


### Progress of the Medical Sciences.

### MEDICINE.

The value of tuberculin was a subject much discussed in the various sections of the British Medical Association meeting at Bournemouth. The President of the Therapeutic section, Dr. Snow, initiated a discussion on "Koch's Treatment," and various papers were read by Dr. Coghill, Sir Walter Foster, Dr. Markham Skerritt, Dr. J. K. Fowler, and others. At the same time "The Etiology and Prevention of Phthisis" was being discussed in the Medicine section, and "The Value of Professor Koch's Method in the Treatment of External Tuberculosis" was being discussed in the section of Surgery. It is not my intention to enter fully into this topic, inasmuch as I could only repeat the observations made in our last number; it was anticipated that the annual meeting might sound the death-knell of tuberculin as a therapeutic agent, and certainly no very enthusiastic remarks were made in its favour. It is to be regretted that the subject was not discussed in a conjoined meeting of the various sections; the numerous discussions held in small meetings could lead to no very definite result, and only partial justice was done to the many and valuable contributions made. The literature of tuberculin is getting to be enormous, but we appear to be only on the threshold of the subject: we have learned that it is not a universal specific and that it is often dangerous, but nevertheless that it frequently gives results far superior to any other drug. We are now only beginning to discover that the drug is a complex one, and that its various components have effects, some of an injurious and some of a remedial character. Dr. Wm. Hunter's elaborate investigation recorded in the *British Medical Journal* of July 25th, and the work of Mr. Watson Cheyne on the action of the chemical derivatives of tuberculin, are the foundation of what may lead to more important discoveries of the future. It has been clearly shown that the fluid is not a vaccine: it contains no micro-organism, it has no vitality, and is not capable of selfreproduction; neither is it an attenuated virus resembling that of anthrax or rabies. The question "What can it be?" is well discussed in the *Annual of the Universal Medical Sciences*, 1891, where it is pointed out that bacteria take from the nutrient gelatin certain elements necessary for their development, and they leave the gelatin in a state of partial chemical decomposition: unstable chemical elements result, these come together, and form new compounds; among these new chemical compounds are the new series of alkaloids called ptomaines, of such unstable chemical nature that we are unable to separate, to analyse, or determine their chemical symbols. The various toxalbumins resulting from the development of the bacillus of tubercle are now being slowly separated and their therapeutic value learned.

We have good grounds to hope that, as the year now ended has shattered the hopes and expectations of those who in an outburst of enthusiasm anticipated too much, so the coming year may give us more exact views as to the nature, the composition, the properties, and the therapeutic value of one of the most powerful agents ever employed in the treatment of disease. It is easy to decry bacteriology, and those who know least of it do so the loudest, but a perusal of the work of this section at the recent Congress of Hygiene and Demography will convince the most incredulous of the increasing importance of the subject in the etiology of disease. We have not yet fathomed the mysteries of tuberculin: at present it may be under a cloud, but light may yet come, and may give some one or other of its derivatives an established position in the therapeutics of the future.

Meanwhile the investigations on Cancer as an Infective Disease bid fair to be of surpassing interest and importance. Mr. Ballance, in his paper on this subject on the second day of the Congress, described various transplanting experiments he, in conjunction with Mr. Shattock, had performed. These had so far given only negative results in transplanting from man to animals, although successful from dog to dog. He pointed out the geographical distribution of cancer, it being especially prevalent along the course of rivers which overflow their banks. In conclusion, he thought there were urgent reasons for believing cancer to be a micro-parasitic disease. Dr. Sheridan Delépine's paper on "Psorospermosis, and its Relation to Malignant Epithelial Tumours" directed attention to a subject on which little is yet known; the author could not prove that the psorosperms are the cause of cancer or even that they are always present, but he had found them in a very large proportion of malignant tumours examined, and they can be easily seen in specimens of malignant disease of the liver in rabbits. Numerous photographs of these bodies were exhibited on the screen by the electric lantern: they are like large oval epithelial cells, but differing in size; they have not yet been cultivated.

The etiology and treatment of diphtheria have received much recent attention; numerous papers were presented to the Congress of Hygiene, and fresh evidence has steadily accumulated showing the importance of the Klebs-Loeffler bacillus in the early development of the disease. A parallel rise and fall of the mortality from typhoid fever and diphtheria in Tournai has been considered by Dr. E. Schrevens to indicate that both diseases are of fæcal origin; and the bacilli of Loeffler have been found to develop, progress, and and extend wherever filth and rubbish of all kinds are stored up. "Of capital importance," says Professor Welch, "is the establishment of the fact that the diphtheritic bacillus develops only locally at the site of infection, and does not invade the tissues or the circulation. It is found only in the diphtheritic pseudo-membrane, and not even in the subjacent mucous membrane. Indeed, it is only the superficial parts of the false membranes which contain the bacilli. The determination of this fact gives at once a clear and decisive answer to the long-mooted question as to the primarily local or constitutional nature of diphtheria. The germ which causes this disease not only makes its first appearance and multiplies where the pseudo-membrane is formed, but it does not even subsequently invade the blood and organs."[1]

It is, however, necessary to recognise at least two varieties of diphtheria, the one originating from the action of the Klebs-Loeffler bacillus, and the other produced by the action of microbes differing from these, with different symptoms and a different pathology. "The Klebs-Loeffler bacillus alighting upon the faucial or other mucous surface, or the skin denuded of its epidermis, obtains there a nidus favourable for its development and propagation, but it does not enter the interior of the system. It is not taken up by the lymph-ducts or blood-vessels and conveyed to the internal organs. It remains localised upon the surface, and produces there the characteristic inflammation. Acting solely upon superficial

[1] *Medical News*, May 16th, 1891.

parts, it cannot in itself produce systemic infection or blood-poisoning, but, as the venomous reptile or the bee secretes a poison which it communicates by its fang or sting, it produces a poison called a ptomaine, which is readily taken up by the lymphatics and blood-vessels and conveyed to every part of the system; and this ptomaine produces the systemic infection or poisoning from which so many of the victims of diphtheria perish." [1]

The observations of Brieger and Karl Fraenkel, those of Klein, of Bates, of Armand Ruffer, and numerous others, are in accord, and tend to show that the bacillary origin of true diphtheria is as well established as most of the accepted theories relating to the origin of the infectious diseases; not only is the bacillus found to be invariably present, but it may be made to produce a disease which has distinguishing characters, especially the production of a peculiar toxine, which brings about certain definite anatomical changes in the interior of the body—a peculiar paralysis and a nephritis, attended by an albuminuria which does not cause dropsy or uræmic poisoning.

As regards the cases of pseudo-diphtheria, the most common form of which occurs during the course of the eruptive fevers, numerous observers are in accord that the special bacillus is absent, but that the disease results from the agency of other microbes.

The specific origin of diphtheria is being more and more abundantly demonstrated, and hence the necessity for increasing cautions with regard to isolation and disinfection. The fact that the disease is to a great extent under control by local treatment in its early stages has led to that contempt, bred of familiarity, which scorns to appreciate the full danger resulting from unquestionable contagiousness. The degree of contagion is limited; just as in typhoid fever, in phthisis, in leprosy, the contagion is well under control; but nevertheless diphtheria must be dealt with on the lines on which all specific poisons are to be limited in their action on the individual and on the community around. Not only is isolation a necessity, but every effort should be made to destroy the contagium *in situ* before the microbes have had time to elaborate their poisons and infect the organism.

The treatment of the toxæmia resulting from the absorption of the poisonous principles produced by the bacilli must be conducted on general principles until some proper antidote to the special toxine has been discovered; meanwhile the

[1] *Annual of the Universal Medical Sciences*, 1891.

time-honoured perchloride of iron in combination with perchloride of mercury has given, and is giving, very satisfactory results.

The revival of an old fallacy as to the therapeutic efficacy of olive oil in expelling large numbers of gallstones has led to much discussion of this topic, and the latest contributions from the Congress for Medicine at Wiesbaden are epitomised in the *Medical Chronicle* (August, 1891). In spite of the inherent improbability that any drug in the alimentary canal can cause the expulsion of gallstones, and in spite of the observations to which I have previously called attention,[1] that the so-called gallstones passed in hundreds or thousands after the administration of several ounces of olive oil are chemically composed of partially saponified oil with only minute traces of cholesterine, there still remains some clinical evidence in favour of the utility of this method of treatment. Possibly the oil may lubricate the passages, and have some mechanical action which may aid in the expulsion of a true cholesterine calculus, but that the oil will do what has been reported of it cannot be admitted, and there is little doubt that it is from the superficial resemblance of the semi-saponified masses of oil to true gallstones that the reputation of the method has been derived.

Durande's mixture of turpentine and ether, in the form of capsule, in doses of twenty to thirty minims, is certainly of clinical value, and the salicylate of soda is also useful; but there can be little question that constant and copious flushing with hot alkaline water is by far the most important means we have of modifying the tendency to the deposition of crystalline cholesterine in the form of calculi. If the patient, the case being a chronic one, can be persuaded to take a dose of four or six ounces of Lucca oil it can do no harm, and in some reported cases it appears to have been of benefit. In one case under my care such a dose was followed shortly afterwards by the expulsion of a small, round, smooth calculus of crystalline cholesterine, and the patient lived several years, but had no recurrence of biliary colic.

In the Pathology section at Bournemouth, Dr. E. Lloyd Jones read a paper "On the Specific Gravity of the Blood in Disease." He appears to have so improved the method of taking the specific gravity of blood that it can be utilised for

[1] *Lancet*, vol. ii., 1881, p. 351.

clinical purposes, and he considers his results to be in every way more reliable than those of the hæmacytometer and hæmaglobinometer. A ready and trustworthy method of estimating the quality of the blood is likely to be of the utmost value, and will materially assist in the classification of our cases of anæmia, in accordance with the views of Dr. Hunter, into cases of defective hæmogenesis or increased hæmolysis, a classification which will aid in the determination of what kind of treatment is likely to be successful.

R. Shingleton Smith.

### SURGERY.

On the subject of the operative treatment of diseases and injuries of nerves, Dr. John B. Deaver has an interesting paper in the *Medical News* of August 8th, and he has reported some cases on which he has operated. The most important of the diseases of the nerves for the relief of which operative treatment may be resorted to are:

I.—Painful affections, including neuralgia, neuritis, and neuroma.

II.—Local spasmodic affections, including such diseases as spasmodic torticollis, facial tic, tetany, and athetosis.

III.—Chronic contractures, including those the result of old traumatisms and those due to hysteria.

IV.—Sclerosis.

I. Cases in which the neuralgia can be traced to a known exciting cause, such as injury of a nerve, or pressure upon or irritation of nerve trunks, and such peripheral irritation as is present when the end of a nerve becomes involved in a cicatrix, offer the best outlook for operative interference. The so-called epileptiform neuralgia, which almost invariably attacks the face and involves any one or all of the three branches of the fifth nerve, is also favourable for operative treatment. This form of neuralgia consists of two main varieties: one in which the pain is accompanied by twitching of the facial muscles, and a second in which pain is the only pronounced symptom. Dr. Deaver reports three cases of this affection in which he removed the superior maxillary nerve with great relief of symptoms; three in which the inferior dental nerve or part of it was removed, with complete relief; and one case of excision of two inches of the long saphenous nerve for chronic neuritis, with perfect recovery. In explanation of the way in which operation does good in neuralgia, Dr. Deaver says it is probable that, in this affection as in locomotor ataxia, the pains may sometimes be due to molecular disturbance, or impaired nutrition of the nerves themselves, or of the posterior root zones, or posterior horns of the cord. Operation probably changes the molecular arrangement of the nerves and nerve-centres, and stimulates nutrition.

In neuritis, according to the stage, we have a more or less high or low grade of inflammatory process. In the chronic stage of many cases, the inflammation has practically subsided and left an exudation and adhesion, the nerve being secondarily degenerated. Operation is useful in two or three ways: (1) By opening the sheath and breaking up any adhesions that may be present; (2) When degenerated, by disturbing the molecular condition of the nerve, and stimulating it to take on regenerative action; or (3) In those cases of chronic inflammation of a nerve in which physiological movements never allow it to be sufficiently long at rest to effect a cure, by removing a portion of the inflamed nerve, and thus getting rid of the constant irritation and giving rest.

Neuromata when found in the continuity of a nerve call for removal, which should be done with great care, to avoid dividing the nerve; if the nerve is divided, the ends should be immediately approximated by suture. When met with in stumps, removal if feasible is also to be practised; and when not feasible, re-amputation offers the only alternative. There is a variety of neuromata known as plexiform: Bowlby has described it as being made up partly of soft connective tissue and fat, imbedded in which are medullated nerve-fibres and white fibrous tissue. Dr. Deaver removed one such five years ago, and it has not recurred.

II. Dr. Deaver thinks the frequent failure of operation in cases of torticollis may be either: (1) Because the spasm may be caused by various muscles which have diverse nerve-supplies, or (2) Because the spasm is sometimes due to irritation reflected from sensory nerve areas to the motor area, as for instance from the major or minor occipital to the spinal accessory, or (3) Because the spasm is due to lesion of cortical or other encephalic centres.

In cases of facial tic, he thinks the frequent failure of stretching of the facial nerve is due to the simple reason that there is a degenerative lesion of the facial nucleus, or to cerebral irritation or degeneration.

In tetany, nerve-stretching may for a time arrest the tremor and twitchings associated with the spastic contractions, but the operation is not usually to be advised.

Nerve-stretching in the treatment of athetosis has not been of much avail, causing only temporary cessation of the clonic spasms and a paretic condition of the affected part. When the paresis passes off, tremor and spasm return. The lesion in these cases is usually situated in the cortex, or near the thalamus in the interior of the brain.

III. In old traumatic cases of chronic contractures of nerves, nerve stretching may do good by relief of the chronic neuritis and loosening of the attachments present. In hysterical contractures, if milder means fail, and the operation does good at all, it is by acting on the spinal centres, or by mental impression.

IV. The usual temporary effect of nerve-stretching in the treatment of lateral sclerosis is, that the spastic condition of the muscles is partially overcome, the tingling in the extremities disappears, locomotion is improved, and the reflexes are not changed; the benefit is not permanent either in this or in posterior sclerosis.

Dr. Deaver thinks that nerve-stretching has had rather a limited range of application, and he suggests that the neurologist and surgeon are justified in acquiring actual experience, by means of clinical experimentation, in other affections than those which have in the past been recognised as fit subjects.

*Nerve Injuries.*—The most common form of nerve injury for which the surgeon is commonly called upon to operate is division. Formerly, tetanus was dreaded as a complication in suture of divided nerve-ends. Since the days of antiseptic surgery, this is no longer feared. Dr. Deaver recommends that suturing should be practised in all cases of divided nerves, and that one or more aseptic silk sutures should be passed through the nerve, neurilemma and tubules inclusive, a short distance from either end, and drawn tight, making apposition perfect. Should there be a loss of substance in the nerve, he suggests: (1) That the cut end of the nerve should be split in such a way that by drawing down the section from the proximal end it can be united to a section from the distal end; (2) Unite the distal end of the divided nerve to a neighbouring nerve; (3) Implant a section of nerve taken from an amputated limb or from a lower animal; (4) That the divided ends be connected with strands of catgut, also to introduce a decalcified bone tube between the ends of the nerve, on the supposition that nerve tissue will be prolonged along the strands of gut or into the calibre of the tube.

I have found it a useful plan in suturing nerves to tie a bit of the sheath on either side, about $\frac{1}{4}$ inch from the proximal end, with separate sutures, then a bit on either side of the distal end in the same way, and then tie together a proximal and distal suture on either side; these form stitches of relaxation, and, if necessary, a very fine catgut suture may be passed through both ends of the nerve to keep them in accurate apposition.

M. Tuffier presented at a meeting of the Sociétè de Chirurgie of Paris, in June, a patient on whom he practised, in March last, excision of the summit of the right lung, for localised tuberculosis. Before operation, he made several experiments. First he attempted through a small inter-costal incision to separate the pleural covering from the lung and get a ligature round it; but although he found it easy enough to detach the pleura above, in front, and behind, it was not so on the inside; and as the thoracic duct and phrenic nerve are intimately adherent to the pleural layer, he was afraid of including them in the ligature, and consequently abandoned all attempts in that direction. Then various questions presented themselves: (1) How would the lung tissue bear a ligature? (2) What is the mode and rapidity of reparation of the loss of substance? (3) What should be feared from the innumerable vessels of the organ? (4) How would the void left in the thoracic cavity be filled? To answer these questions satisfactorily, he resected the summits of the lungs of several dogs, and the result of his experiments was that the wound rapidly healed and the void was quickly filled by expansion of the parenchyma, provided the part resected was not too considerable. The man operated on was 25, of stout build, and enjoyed good health until two months before operation, when he was attacked with cough, night-sweats, and tuberculous laryngitis, for which he entered the hospital. At the apex of the right lung, dulness, respiration rude and jerky, expiration prolonged, and dry crepitation on coughing were noted; the left lung was healthy.

Through an incision in the second intercostal space, the lung was reached and the pleura separated; the apex of the lung was then drawn forward with special flat forceps, a ligature thrown round it, and the pedicle fixed to the periosteum of the second rib. The resected part was then examined, and in its centre was found an induration the size of a small walnut, with tubercles round it in which bacilli existed. The man made a rapid recovery, and a minute auscultation of the lung showed that the respiratory murmur, though a little weakened, was present throughout the whole organ, without revealing the slightest trace of a pleural effusion.[1]

Mr. James E. Thompson, in the *Medical Chronicle*,[2] quoting from Krecke, gives particulars of lung affections amenable to surgical treatment. Gangrene of the lung is specially suited to surgical measures: the stinking, loathsome expectoration is characteristic, and spontaneous cure is hardly to be expected. In gangrene with empyema, after letting out the pus, portions of one or two ribs should be resected, and a careful examination made of the surface of the lung; if one finds anywhere on the surface of the lung an irregular indurated spot, or soft focus, it should be laid open carefully with a thermo-cautery. Where empyema does not exist, the diagnosis is very difficult; the lung should be punctured again and again until one hits on some characteristic foul sanious fluid or putrid gas. In one case the lung was punctured as many as fifteen times, and at last a foul, brown, sanious fluid was procured; two ribs were resected, and a gangrenous focus opened. Iodoform gauze was stuffed in the wound; the patient improved for a time, but died shortly after from mischief in the other lung. The *post mortem* examination showed that the gangrenous patch had almost healed. Notwithstanding the high mortality, he advises operation in all cases. Slawyk had 14 cases and 8 recoveries; Seitz had 19 cases and 4 recoveries, while 4 more improved considerably.

In cases of abscess of the lung, the same rules hold good. Slawyk, out of 13 cases, had 3 recoveries; Seitz showed 11 cases, with 5 cures.

The treatment of bronchiectatic cavities is a little *sub judice*, because in addition to one large cavity there are often a number of smaller ones in various parts of the lung. Krecke operated on one case in which acute fever developed in the course of a chronic lung complaint, with dulness over a small area: the needle revealed pus there and none elsewhere; unfortunately, when the rib was resected, the lung collapsed, and he was unable to open the spot. The autopsy showed the presence of a bronchiectatic cavity.

In treating tuberculous cavities, the presence of further tuberculous processes has tended to frustrate all advance. Surgical interference ought only to be undertaken when single cavities are known to exist. He cites the case of a man

[1] *Therapeutic Gazette*, August 15, 1891. [2] August, 1891.

æt. 33, in whom a cavity gradually developed in the right lung. Suddenly septic fever, with rigors, intervened, and blocking of the communication between the cavity and the bronchus was diagnosed, with accumulation of fluid behind the obstruction; parts of two ribs were excised, and the pleural cavity was found obliterated at this spot; a pair of blunt forceps were thrust into the cavity through the lung tissue, and a free passage made for the pus; the cavity was cleared out and stuffed with iodoform gauze. Fever disappeared, and the condition of the patient became very much better.

It is in such cases as these that operation is certainly indicated. Krecke lays stress on some points of operation detail: (1) Always use a blunt instrument to perforate lung tissue; (2) In deep-seated foci, the incision ought to be made with a thermo-cautery; (3) Use tampons of sterilised gauze for plugging a cavity; (4) All washing of cavities should be discountenanced, lest the fluid should trickle into the bronchi and cause irritation.

W. J. Penny.

### GYNÆCOLOGY.

For the last four years Electricity has been more or less on its trial. By the majority it has been discarded as absolutely useless; the minority, who have really taken the trouble to thoroughly work out the subject, act otherwise.

There would be no object in entering into the various details of the subject here, as these can be learned from any ordinary text-book; but it is rather astonishing to read the opinions of writers who do not seem to understand the difference between the positive and negative currents, and apparently apply the treatment in a most haphazard manner, and then blame it because the patient is not cured.

As in everything else it is a matter of experience, and we must not expect to cure every disease to which woman is subject, or imagine that every particular class of ailment is certain to be benefited by it. It can be laid down with absolute certainty that the treatment has never, in the hands of those who understand it, done any harm or produced any serious symptom; and in a very large number of cases it has brought such relief to the unfortunate sufferers that we are amply justified in keeping it in our list of valuable remedies. In the first place, it is necessary that the person who intends to use this treatment should not so much have a knowledge of electricity as of diseases of women, otherwise it is very easy to see that his results might not only be disastrous but even fatal; and there is no doubt that electricity has been applied in numberless cases where it was not in any way admissible.

In that large class of cases of myoma uteri its effect appears to be almost incredible; and when one is told by the patients themselves that the bleeding is much less, the pain has almost disappeared, and they have had to take in their dresses by five inches and a half round the waist, and that they really feel now life is worth living, there is a clear answer to those sceptics who say the treatment is useless, and that nothing short of removal of the ovaries or hysterectomy will be of any benefit. Although in competent hands these operations are almost void of risk, yet a very small percentage die; it is therefore right to give trial to a treatment which, without fear of contradiction, can be declared absolutely without any danger. In this class of cases the positive pole has been used within the uterus with all the necessary precautions that it is absolutely essential to take if success is to be attained.

It should, however, be always remembered that if myoma uteri is complicated by disease of the uterine appendages, electricity is almost worse than useless, and if it is persisted in, disappointment or worse may follow. Such cases, no doubt, should have the appendages removed, as we would remove any other part of the body (if diseased) that was possible. Removal of the appendages (if healthy) will not always check the hemorrhage in myoma uteri, and cases have already occurred in which the electrical treatment has been brought into use with great success when the above operation has altogether failed to improve the patient's condition.

In subinvolution of the uterus this treatment produces a most marked improvement, and it is only necessary to employ it as a rule on three or four separate occasions.

Patients who suffer from dysmenorrhœa are only too eager to try this remedy, and apparently not without reason, for if the symptoms are due to a narrow condition of the cervical canal, the intra-uterine use of the negative pole causes at once a dilatation, which remains permanent. I may also mention that pregnancy has quickly followed this treatment in a few cases of women who were married and sterile for years suffering from dysmenorrhœa. In the case of one woman who had been in this condition for three years, this occurred within eight weeks.

In many cases of pelvic pain, associated with a neurotic mental condition, the Faradic current appears to produce alleviation, but at present I am not inclined to attach so much importance to the local effect, but more to the fact that to a large number of cases this is something quite new in treatment. It is also very easy of application, and can be entrusted to a nurse.

In treating cases of chronic dyspepsia or obstinate vomiting, one should always bear in mind that there may be some uterine trouble existing, and to which one's attention may never be directed on account of the real lesion being so far from the apparent symptoms. These are really referred nerve symptoms or reflex phenomena, brought about by means of the vaso-motor system, the centre of which is in the medulla and grey matter of the cord. Chronic endometritis or some slight retroflexion of the uterus may be the cause of these referred neuroses, and the introduction of a properly fitting pessary or some local application to the cervix and os uteri will frequently act as a charm, and perform an almost miraculous cure on a chronic invalid.

Dr. Herman writes of fungous endometritis in two forms (1) the hyperplastic; (2) the polypoid. Bimanually the uterus is found to be slightly enlarged, and the cavity of the body is found to be expanded if the cervix is dilated. The chief symptoms are hemorrhage, with a watery discharge in the intervals. The best treatment is to scrape away the diseased mucous membrane with a curette and then apply caustics, and, above all, to use the curette in the polypoid form. This treatment is as a rule followed by complete recovery, and subsequent fertility is not interfered with.

Whether some of these cases have any relation to malignant disease is at present in great doubt; at any rate, some of them ultimately die of cancer, when apparently the original disease has for a time been in complete abeyance; and the explanation that naturally suggests itself is that the original diagnosis was a faulty one, but very competent judges assert that occasionally malignant disease does develop out of changes which at first had no such character.

Another most important and unfortunately most common form of endometritis is that produced by gonorrhœa. It has always been asserted that this starts first in the vagina, and then affects the cervix and later on the Fallopian tubes; but it is now quite an open question whether it is not possible for a latent form of gonorrhœa to be started by communication in the cervix itself without any preliminary vaginal symptoms. If we believe that every case must have its origin in the vagina, then we surely ought to adopt some much more radical and effectual treatment in the early stages with a view of preventing the terrible consequences of the spread of the disease upwards. The ordinary syringe with some lotion or other is not only useless but absolutely harmful, and must help to carry the mischief onwards. Unfortunately we are forced to the conclusion that the disease in some cases starts in the cervix itself, and the ovaries and tubes are already affected before we are even consulted; and this apparently occurs where there has been no history of the gonorrhœa itself for perhaps five or six years before marriage, so that we are beginning to believe that the disease may lie dormant for almost an indefinite period, and a woman is more likely to be injuriously affected by a previous gonorrhœa even than by a previous specific history. When once this mischief has spread to the ovaries and tubes, it is really incurable, and they might as well be removed, for the patient must now be permanently sterile. Moreover, it is the shortest cure for these chronic invalids.

There is hardly anything more difficult to diagnose than malignant disease of the interior of the uterus. Of course anyone can recognise a typical case, but then unfortunately it is generally too late. The ordinary medical attendant should always view with the greatest suspicion any irregular hemorrhage, especially if it should occur after the menopause, and he should never be satisfied until he has absolutely excluded the diagnosis of malignant disease. We must not expect to meet with the severe lancinating pain due to the exposure of nerve-endings by ulceration, or the offensive discharge caused by the advancement of the necrotic changes, or yet the cachexia due to septic absorption. Again, we must not expect that the hemorrhage should be profuse: occasionally it may be only a slight escape of blood after some undue exertion, and the patient may think it is simply some slight return of her menses. We ought to be able to differentiate accurately between malignant disease, hyperplastic endometritis, and sloughing fibroid, and it must not be forgotten that a small submucous polypus will often give rise to symptoms that closely resemble those of malignant disease.

The only certain way to settle the diagnosis in these doubtful cases is to dilate the cervical canal (I think preferably with tents), and then explore the uterus with the finger and the curette.

Numerous cases have been recorded in which death has ensued from uncontrollable uterine hemorrhage, and on the *post mortem* table it has been found to be due to a polypus, submucous fibroid or retained product of conception, which could easily have been removed, and thus the patient's life been saved. If we insert a sponge tent into the cervix uteri, we at any rate arrest the hemorrhage for the time, and the danger of any reflux through the Fallopian tubes is merely visionary. The unscientific expedient of plugging the vagina should hardly ever be resorted to: it is practically useless, and does not assist us in any way to a further diagnosis.

W. H. C. Newnham.

### OPHTHALMOLOGY.

Nicati, in the *Archives D'Ophthalmologie*, has recently very fully described the "Gland of the Ciliary processes" which secretes the aqueous humour. The essential glandular structure is the epithelium, which extends from the ora serrata behind, to the base of the iris in front; completely covering the ciliary processes and the recesses between them. Its secreting surface is estimated to cover an area of six centimetres square.

The epithelium is of two distinct kinds, in two layers. The external is the direct continuation of the pigmented hexagonal outer layer of the retina, the cells of which become modified as they pass anteriorly: the internal layer consists of prismatic cells compressed together at their sides, so that they stand high or deep, rather than wide; they are modified from and directly continuous with the inner layers of the retina, more particularly with the ganglion cells. The rods and cones, together with the external granular layers, do not extend into the ciliary region but cease at the ora serrata.

Treacher Collins has more thoroughly examined the exact character and arrangement of these cells by a method of bleaching-out the pigment. He found the uveal pigment layer from the root of the iris to the ora serrata to be composed of a single row of somewhat flattened cells, projecting from the outer surface of which were numerous processes, each composed of a group of cells, tubular in arrangement, or even pear-shaped, and containing a small central lumen. These tubular processes he considers to be nothing else than secreting glands concerned in the elaboration of the aqueous humour and the nutrient fluids of the vitreous.

Nicati proceeds to consider the special anatomical arrangement of the blood-vessels. Outside the epithelium, and scarcely separated from it by a very thin reticulated tissue, lies the dense network of large capillaries (the anterior prolongation of the chorio capillaris) which supplies alimentation to the gland. He describes the chorio capillaris—first where it lies posterior to the ora serrata—as consisting of three coats: (1), the middle one, the capillary layer, separated on the inner side from the retina by, (2) the homogeneous membrane of Bruch, and on the outer side from the arteries and veins of the choroid by (3) the intervascular membrane of Sattler, which is perforated by the blood-vessels, to reach the capillaries. These two membranes form a complete sac lining the interior of the eyeball, from the optic nerve entrance forwards, and containing within it the capillaries. Both membranes are impermeable, so that any secretion which passes from the capillaries is retained within the sac and can only escape by passing forwards along it to the ciliary processes. Here in front of the ora serrata the arrangement is much modified: the capillaries become largely developed, while the membrane of Bruch, hitherto impermeable becomes so attenuated as to allow of easy exudation from the capillaries into the epithelial cells of the ciliary processes, whence the aqueous is secreted. Experiments show that the whole of the chorio capillaris participates in the elaboration of the aqueous fluid.

Nicati appears to hold the opinion that the membrane of Bruch prevents communication between the choroidal capillaries and the retina, and that the view usually held that the retina is nourished by the choroid is inaccurate.

Some facts may be quoted on the other side. The hyaline lamella which separates the retinal pigment from the choroidal capillaries is very thin, so that they are almost, if not quite, in contact: the vessels increase in number very considerably in the neighbourhood of the yellow spot, and at the fovea centralis vascular development attains its maximum: these parts of the retina where nutritive changes are most actively going on, necessitated by the photo-chemical processes in the cones, receive scant supply from the arteria centralis retinæ. Regeneration of the retinal purple takes place from the choroidal surface rather than from the side of the retina. Certain facts in comparative anatomy and in pathology also show the dependence of the central parts of the retina upon the choroid.

The arteries concerned in the blood-supply to the uveal gland are the long posterior ciliary, which pass forward without entering the choroid and send numerous recurrent branches into the ciliary body. The short posterior and the anterior ciliary arteries are not concerned in this distribution. The veins run chiefly inside the muscle fibres towards the equator of the eye to join the vasa vorticosa. Muscular action thus influences the venous outflow rather than the arterial supply.

The aqueous humour leaves the gland by three main channels: (1), Petit's canal *(le canal godronné)*; (2), the posterior chamber; and (3), the inner projections of the ciliary processes, and the suspensory ligament. It may be here mentioned that other recent investigations show that the zonula is not a membrane so much as a meshwork of fibres which are attached to the whole free surface of the ciliary processes, excepting their inner rounded projections, and particularly to the recesses between them; some of the fibres appear to arise from within the epithelial cells. At the lens they pass to the equator as well as to the anterior and posterior surfaces.

The anterior chamber forms the reservoir for the aqueous conveyed to it by the channels just mentioned; from this no outlet is possible through Descemet's membrane, but absorption probably takes place through the lacunæ and spongy channels of the iris, and through the spaces of Fontana; the current goes along through the perivenous lymphatics, and finally empties into the blood stream of the anterior and posterior ciliary veins.

The tubular glands of the ciliary processes have been affected by tumours, adenomata and glandular carcinoma; and like secreting glands elsewhere they are very liable to catarrhal inflammation, a condition which is clinically known as serous iritis, but which might be better named serous cyclitis. Cases are found in practice which bear to ordinary iritis much the same relation that pleurisy does to pneumonia. The proper tissue of the iris is not primarily implicated but rather its uveal layer, and perhaps also very frequently the glands of the ciliary body. The classical symptoms of iritis are absent, there is neither discoloration nor narrowing of the pupil; no photophobia nor lacrymation; circumcorneal hyperæmia may or may not be present. The condition is extremely insidious and may have recurred at longer or shorter intervals without any anxiety on the part of the patient, who fancies he has had a slight cold in the eye. Meanwhile posterior synechiæ and cloudiness of the media come on. The affection is most common in women from thirty to fifty, and depends neither upon syphilis nor rheumatism, and the remedies often useful in iritis do no good. Atropine is of little service, but a large iridectomy usually effects a cure.

The condition of the iris, and of the secretory channels of the ciliary gland, as affecting the secretion and excretion of the aqueous humour, is a point of much importance in the pathology of many faulty conditions of the eyeball, *e.g.* glaucoma, detachment of the retina, and irido-cyclitis.

Thus the outflow of the aqueous may be obstructed by various abnormal conditions of the iris, its congenital insufficiency, its progressive atrophy from senile rheumatism or gouty changes, or by the reduction in area of its absorbing surface due to any form of mydriasis, or by inflammatory engorgement of its tissues.

Hypodermic medication, not very generally used excepting in the case of a small group of remedies, has received a considerable stimulus through the introduction of tuberculin. In addition to such local effects as are produced by cocaine and morphia may be mentioned the action of strychnia in optic nerve failure when the drug seems to be distinctly more efficacious if injected in the region of the orbit. At the recent meeting of the French Ophthalmological Society the subconjunctival injection of remedies for the treatment of various maladies in the eyes was recommended by M. Darier. He reported very favourably of this procedure, to which he has given a good trial in all kinds of cases, more especially in those of an infectious nature.

He injects $\frac{1}{20}$th of a milligramme of sublimate every three or four days by means of a Pravaz syringe rendered aseptic by $\frac{1}{20}$th carbolised glycerine, and he asserts that in cases of acute iritis, whether specific or no, in gumma of the iris and in keratitis, no other form of medication gives such favourable results in so short a time. He also says that good results have followed its use in neuritis, and in inflammations of the retina and choroid.

Abadie also speaks strongly in favour of this treatment. He has abandoned intra-ocular injections (*Bristol Med.-Chir. Journal*, March, 1891) in favour of the subconjunctival method. I have found it of value in inflammations of the episcleral tissue, but have no experience of it as affecting intra-ocular diseases. It is certainly not attended by risks such as those involved in intra-ocular injections. Much irritation sometimes follows the use of the sublimate, but this tendency is lessened by using fresh solutions in distilled water.

Trichloride of iodine is recommended by Pflüger as being equally efficacious as a bactericide, and much less irritating. He has experimented upon the direction of the currents out of the subconjunctival tissue. Injecting fluorescine here or into Tenon's fascia, he found that it travelled along the cornea, gradually spreading entirely over it. It passed into the uvea, into the suprachoroidal lymph space, and into the vitreous, especially the anterior portion, and even into the lens. The lens retained the change of colour many days after it had disappeared from the other parts of the eye, and showed in a beautiful manner the nutritive zones which Magnus has described. The retina and the optic nerve remained throughout uncoloured.

Nieden, who is a strong advocate for the use of the galvano-cautery in various forms of corneal ulceration, finds a 2 per cent. solution of the potassium salt of fluorescine a valuable aid in the selection of cases suitable for operation, and in forming a good judgment how much tissue requires removal the peculiar green tinging of the parts bringing them into view of the naked eye wherever fresh inflammation may have occurred.

Nystagmus is found in early life always associated with dulness of sight, due either to defective transparency of the eyeball (leucoma corneæ and cataract, or aniridia and albinism) or to faulty development of the visual nerve-tract, so that a perfect retinal impression is impossible, and *the natural stimulus to steady fixation of the eyeballs is absent.* In later years, lesions of the cerebellum and multiple sclerosis give rise to similar clonic movements of the eyeball, because they have damaged the oculo-motor centres and tracts, and *disturbed the muscular equilibrium.*

In Miners' nystagmus there would appear to be present *a disturbance of the muscular equilibrium* from overstrain of the eye muscles, which maintain an unusual position for many hours together in the unnatural attitudes which "coal-getters" assume at their work—a form of "functional impotence" analogous to those diseases of which writer's cramp is the type; there is, moreover, only very imperfect illumination of the black groundwork around, and little if any *stimulus to the steady fixation of the eyeballs.*

Snell has for many years past paid much attention to this subject, and he considers that the prime cause lies in the constrained attitude of the body and the oblique inclination of the head and eyes. He has seen about five hundred cases, and has notes of 120; of these latter, 112 regularly worked on their sides in low seams ("holeing"); 3 often worked as holers, and the other 5 were all engaged in duties necessitating the oblique upward position of the head. He has found that recovery follows relief from duties requiring this constrained position, although the men still work under the usual condition of defective lighting. Dransart also says that 90 per cent. of the cases he has seen were in miners who had worked lying down in galleries inclined 20° to 45°, and not more than a yard high. The nystagmic movements cease while the patient looks downward, and are exaggerated by his looking obliquely upwards. The principal factor in causation he considers the strained upward and oblique position of the eyes, together with the defective lighting.

Tatham Thompson, on the other hand, finds that the South Wales colliers, who work "house coal" in an awkward and constrained position, many holeing, are but slightly affected by nystagmus; while those cutting "steam coal," with, as a rule, plenty of standing-room, are much affected: these latter use the safety lamp, while the "house coal" workers use the open candle. He considers the faulty light of the safety lamp is an important if not the essential factor in causation, and with this opinion many men of experience in the Welsh pits concur. Pitmen have long blamed the Davy lamp for this disorder.

Dransart, who is the authority in France on this subject, found in 179 serious cases examined as to their dependence on the kind of lighting that 92 used the safety lamp and 87 the naked light. He agrees with Snell that faulty position is the main factor (as does Nieden in Germany), but thinks where the seams are high any cases of nystagmus will be slight or latent, and more frequent and grievous where the seams are low.

Dr. Hudson of Redruth once said that he had never known any case of nystagmus in the Cornish mines, excepting in men who had acquired it in the North. In Cornwall they never work more than eight hours at a shift, often less, and with naked lights, which give better illumination. The peculiarly strained abnormal position and bad lighting are certainly both factors in causation, probably aggravated by overwork and bad air. A good safety lamp of modern construction, kept clean, gives as good a light as an average candle. Illumination underground cannot be excellent, but it might be improved. Dr. Makeig Jones says that nystagmus is much more common in winter than in summer, and that it is found in all conditions of underground workers.

F. R. Cross.

